# Exposure to Self-Directed Violence: Understanding Intention to Help and Helping Behaviors among Adolescents and Emerging Adults

**DOI:** 10.3390/ijerph18168606

**Published:** 2021-08-14

**Authors:** Victoria Banyard, Kimberly J. Mitchell, Michele L. Ybarra

**Affiliations:** 1 Center on Violence against Women and Children, Rutgers School of Social Work, Rutgers the State University of New Jersey, 123 Church Street, New Brunswick, NJ 08901, USA; 2 Crimes Against Children Research Center, University of New Hampshire, Durham, NH 03824, USA; kimberly.mitchell@unh.edu; 3 Center for Innovative Public Health Research, 555 N. El Camino Real #A347, San Clemente, CA 92672, USA; michele@innovativepublichealth.org

**Keywords:** self-directed violence, bystanders, suicide prevention

## Abstract

Exposure to self-directed violence (SDV) is a public health issue. Prevention trains third parties to identify SDV risk and provide help. However, we know little about the range of help provided to those who engage in SDV. The current study used a cross-sectional online survey of 1031 adolescents and emerging adults to learn about their SDV exposure, intent and attempts to help, and barriers to helping. Most participants reported SDV exposure, commonly by a peer, and provided help. Regression analyses showed that intent to help was predicted by social norms and having knowledge of resources, and such knowledge (but not social norms) was also related to actual helping behaviors. Qualitative analysis of short open-ended questions on the survey documented a range of barriers to helping. Findings support but also encourage revision of theoretical models of helping upon which prevention programs are based.

## 1. Introduction

Self-directed violence (SDV), defined as any intentional act that can cause injury to one’s self, including death [1], is a significant public health issue. Youth and emerging adults are a particularly at-risk group; although uncommon in childhood, deaths by suicide, suicide attempts, and self-injurious behavior all increase dramatically in the early-to-middle teens and continue to rise until the mid-20s [2]. Global estimates suggest lifetime rates of suicide attempts to be 6% among youth, 18% for lifetime suicidal ideation, and 22% for non-suicidal self-injury [3]. Importantly, many youths are also exposed to the SDV of others, including peers and family members. For example, 12% of a nationally representative sample of youth aged 10 through 17 (4% aged 10–12, 13% aged 13–15, and 21% aged 16–17) said that someone close to them tried to die by suicide [4]. Given the negative psychological [5] and academic impacts of exposure to SDV (including trauma symptoms and thoughts of self-harm) on people who are close to the person experiencing SDV [4,6], understanding the experiences of bystanders is critical but lacking in this context (Helpful third parties are referred to as bystanders or actionists in interpersonal violence literature, defenders in bullying literature, and gatekeepers in the field of suicide prevention. In the current paper, we use the term bystander or active bystander.). To address this gap, the current exploratory and descriptive study examined rates of exposure to suicide attempts, ideation, and non-suicidal self-injury (NSSI) in a national sample of adolescents and emerging adults. The study was crossed-sectional and multi-method, using an online survey to collect quantitative and brief qualitative information. Findings will illuminate the range of exposure young people reported, overall intentions to help, barriers to helping, and the reported actions that were taken.

### 1.1. SDV Exposed Individuals as Potentially Helpful Bystanders

Exposure to SDV has been explored in several ways. One is research on disclosure, a body of work that focuses on people who attempt suicide and whether they seek help or tell others about their intent [7,8,9,10,11,12]. For example, some findings show that disclosure to parents facilitates help-seeking and coping but that while peers are perceived as supportive, disclosure to peers may decrease social support over time [12]. Another body of work seeks to understand suicide survivorship and the impact of knowing someone who died by suicide [13] in order to provide response resources and service to support, especially those who were strongly affected by a loss. Recently, an additional focus has been on third-party intervention by people, especially adults, who may see warning signs for SDV and provide resources to prevent it as helpful bystanders [14]. Indeed, a growing number of SDV prevention programs train adults and peers to be more proactive bystanders when they see someone who may be at risk for self-harm [14,15,16]. Such programs have mainly focused on adults in professional roles, such as teachers. Many have demonstrated positive changes in knowledge and attitudes about suicide but have had less success in changing behaviors (i.e., actual use of the skills learned to help others) in real-world settings [16,17,18,19,20]. More recently, youth are being trained to change social norms that impede help-seeking and to provide help to reduce the risk of suicide and self-harm among their peers [20]. This is important given that not only are adolescents and emerging adults a high-risk group for suicide and suicide exposure, but youth at risk for SDV are most likely to tell peers who then become potential bystanders [21,22]. We need a better understanding of the characteristics that promote impactful SDV prevention behaviors when someone receives disclosure or notices SDV risk. This includes a more complete understanding of youth’s attitudes toward helping, what youth try to do, and what barriers they experience when trying to help. This knowledge could inform more effective SDV bystander prevention programming. The current study looks to fill this need.

### 1.2. Understanding Models of Bystander Intervention: Who Helps?

The situational-cognitive model of bystander behavior was developed to explain responses to risk for interpersonal violence but has broader applications [23] and has begun to be applied to SDV gatekeepers as well [14]. The theory is grounded in Latane and Darley’s [24] situational model, which described aspects of the local context that could inhibit intervention (i.e., the presence of other passive bystanders who promote a diffused sense of responsibility and a norm that action is not needed). When adapting this model for violence prevention, researchers highlighted the importance of understanding barriers (impediments to noticing, taking responsibility, and acting) during the unfolding of the situation [25]. Researchers also recognize that bystanders bring with them more distally instilled attitudes related to their social location and life experiences that can affect how they think about the immediate situation when there may be opportunities to help [23,26]. For example, perceptions of social norms related to helping are strong correlates of action among adolescents and adults in interpersonal violence contexts [27,28]. The intent to help is an important intermediate attitude variable linked to helping in violence prevention as well [29]. The situational-cognitive model has been adapted to include these variables but has been more thoroughly explored in relation to interpersonal violence such as bullying than SDV [23].

Conceptual models developed and adapted to understand interpersonal violence need further empirical examination in relation to SDV [14,30,31]. Studies of college students who helped peers who were at risk for suicide found that intent to help was explained by attitudes such as perceptions that social norms supported the action and that intervening was not difficult, knowledge of resources, and confidence [32,33]. Further, a study of adults trained to intervene to prevent SDV found that greater confidence and self-efficacy were related to higher intent, and greater intent was related to a higher likelihood of actually questioning and referring an at-risk person [34]. Interestingly, a vignette study among emerging adults found that those with prior exposure to suicide were more likely to say they would get help from an adult if a peer disclosed, and a more ambiguous SDV disclosure vignette produced greater intent to talk to the at-risk peer themselves rather than involve adults—especially among youth with their own history of suicidal behavior [22]. This work suggests that further research should include variables within individuals (attitudes such as social norm perceptions) as well as aspects of the situation, such as the type of SDV that the bystander becomes aware of.

Demographic factors (e.g., gender identity, sexual identity, race, and ethnicity), which may also intertwine with exposure to racism and risk of health disparities [35], have been linked to disclosure of SDV and might also be related to helping [36]. For adults, one’s profession (e.g., holding a position in a helping role) is associated with the intent to help an individual at risk for SDV, with more helping among those who see their job description as including such helping actions [14,32,33]. These studies support the situational-cognitive model but have mainly focused on adult samples and have measured intent to help as an outcome rather than actual helping behaviors. Research is needed to examine whether the importance of these variables can be replicated in adolescent samples, to explore bystander helping across a range of SDV situations (suicide attempts, ideation, and NSSI), as well as to study actual helping behaviors in relation to intent to help. Indeed, several studies found age differences related to suicide exposure and risk factors, suggesting the need to examine potential developmental differences. Pre-adolescents report suicide exposure at lower rates than early adolescents, who, in turn, report less than older adolescents [37]. This pattern was also shown for youth exposure to websites related to SDV [38]. These differences in rates of exposure create different opportunities for helping, yet we know little about how that might translate into qualitative differences in how helping happens. In the interpersonal violence literature, for example, we know that bystander training seems to be less effective for older adolescents, which is another line of evidence that correlates and models of helping may change over time for youth and emerging adults [39].

### 1.3. Research Questions

To address the noted gaps in the literature, we will examine the following research aims using data from an exploratory cross-sectional online survey that collected both quantitative and open-ended qualitative data:

**Research Aim 1.** Determine how often adolescents (aged 13–17) and emerging adults (aged 18–23) are exposed to different types of SDV, how exposure may vary based on social location characteristics (e.g., age, race and ethnicity, sexual and gender identity), and describe different characteristics of SDV exposure (e.g., how long ago it happened, relationship with the individual who attempted SDV).

**Research Aim 2.** Test whether greater intent to help is related to perceptions of more supportive norms, greater awareness about resources, and perceptions of a participant’s access to social support (generally and specifically related to SDV concerns).

**Research Aim 3**. Describe the types of self-reported helping behaviors adolescents and emerging adults engage in when they become aware of SDV as well as barriers to helping that they face.

**Research Aim 4**. Test whether greater odds of actual bystander behavior are found among participants with greater intent to help, more positive social norms perceptions and greater resources, such as support.

The answers to these questions will help identify important leverage points for mobilizing helpful bystanders through prevention training that can be included in new prevention initiatives.

## 2. Method

The Exploring Your YOU-niverse Study is a series of independent national surveys of youth and emerging adults. This most recent one investigated exposure to self-directed violence. A cohort of 1031 youth and emerging adults (aged 13–23 years) was recruited between 27 November 2020 and 4 December 2020. Table 1 provides details of the demographic characteristics of the sample.

Participants were recruited through online advertisements on Facebook and Instagram. Facebook allows targeted ads based upon age and sex. The ads encouraged youth and emerging adults to ‘have their voice heard’ and ‘make a difference.’ Survey aims were not mentioned to reduce self-selection bias based upon interest in a particular topic. Those interested clicked on the online advertisement, which linked them to a secure survey website. This first page provided a study description and screening questions to determine eligibility. Those who were eligible (i.e., 13–23 years of age, living in the United States, English speaking) were then asked to read an assent/consent form and to indicate their willingness to participate in the survey before continuing. A waiver of parental permission was granted because requiring parental consent could potentially place youth in situations where their sexual experiences and/or sexual attraction could be unintentionally disclosed to their parents. Appropriate mechanisms were in place to protect the participants, such as localized referrals to mental health supports.

Participants were given a USD 5 incentive as an Amazon gift code for completing the survey. Ineligible participants were directed to a web page that included links to general resources for youth (e.g., https://youngwomenshealth.org, 4 June 2021). To promote a diverse sample, demographic quotas were identified. Once the targeted number of participants in a particular group had been achieved (e.g., aged 13–17, cisgender girls), subsequent youth in this group who were otherwise eligible were deemed ineligible. The protocol was reviewed and approved by Pearl Institutional Review Board (Study ID 19-CIPH-101 approved 18 November 2020).

### 2.1. Measures

The measurement of bystander behavior for SDV among the youth population is in its infancy; most of the existing scales focus on college students. As such, several measures were adapted for the purposes of the current study. This was carried out by drawing from these existing scales and informed by the authors’ experience with bystanders of interpersonal violence. Some of the most widely used existing measures are drawn from the work of Aldrich and colleagues and their Willingness to Intervene Against Suicide Questionnaire [40]. We started with their intention to intervene items and made the following adaptations: (1) four were dropped due to our focus on positive helping behaviors (e.g., “Do nothing it was none of my business”); (2) some items were reworded to be meaningful for youth and non-college students (i.e., removed references to RA and campus); (3) item language was simplified for younger adolescents (e.g., “Give the suicidal person the space he or she needs time to heal” was changed to “Give the person time to get better”); and (4) removal of the term “suicidal” person throughout to be applicable to different forms of SDV. We also included forms of help-seeking from adults and others, as noted by Wyman and colleagues [20]. Further, once we derived our final list of items for bystander intentions, we paralleled these same items across the social norms and actual bystander behavior scales, which is the convention in the interpersonal bystander literature [41]. A final noteworthy adaptation is our focus on close friends as the reference group for the social norms items given this is the most important normative source for this age group in the current study [42]; this is in comparison to Aldrich and colleagues, who included only three social norms items but repeated the items across several reference groups (close friends, people at school, community, and family) for each.

#### 2.1.1. Exposure to Self-Directed Violence

Participants were asked about three different types of lifetime exposure to self-directed violence [43,44]: suicide attempt, suicidal ideation, and non-suicidal self-injury (NSSI). Specifically, participants were asked:

(1) “Has someone close to you ever tried to kill him or herself on purpose (like by shooting or cutting him or herself, or taking too many pills or drugs)?”

(2) “Now thinking of situations where someone was thinking about, considering, or planning to kill themselves. Has someone close to you ever thought about killing themselves but did not make an attempt?”

(3) “Now thinking of situations where someone was hurting themselves on purpose without wanting to die, like cutting or burning. Has someone close to you ever hurt themselves on purpose without wanting to die, as far as you know?”

A positive response to each of these questions was followed by a series of follow-up questions about a specific individual. Participants were first asked about the number of people they knew struggling with each type of SDV. If participants reported knowing more than one person in each of these situations, they were asked to answer questions about the most recent person who did this. We also asked questions to make sure unique individuals were being talked about across the three types of SDV to avoid duplication of situation characteristics. With a few exceptions, which we will note below, the follow-up questions were the same for each type of SDV exposure. Thinking of a specific individual, participants were asked the following questions: their relationship with the person, how long ago the incident happened, and how they learned about it. For suicide and suicide attempts, we asked when they found out about the suicide or attempt (before or after it happened); we also asked whether the person died by suicide (and if not, whether they went to the hospital). For suicidal ideation, we asked whether the person had taken steps to carry out a suicide attempt, such as picking a method; and how sure they were that the person might try to die by suicide. For NSSI, we asked how sure they were that this person was hurting themselves on purpose.

#### 2.1.2. Social Norms

Participants were asked how much they agree or disagree that their closest friends think it is a good idea to get help for someone who wanted to hurt themselves. Eight specific behaviors were queried that paralleled the bystander behavior and intent items described below: (1) talking to a helpful adult such as a teacher, counselor, or parent for advice; (2) talking to a friend about their worries; (3) contacting a crisis hotline for help; (4) giving the person time to get better; (5) telling the person they are worried about them; (6) encouraging the person to talk to their family; (7) encouraging the person to contact a hotline or get counseling (note that this question was separated into two for the bystander behavior questions); and (8) telling the person they matter. Response options ranged from 1 (*strongly disagree*) to 4 (*strongly agree*). Missing data were no larger than 1% for each variable and recoded as the item mean. The reliability for the entire scale was adequate (α = 0.75).

#### 2.1.3. Bystander Intent

Participants were told: “Now we have some questions about what you would do, if anything, if you knew someone who wanted to hurt themselves on purpose. By ‘hurt themselves on purpose’, we mean wanting to kill or injure themselves in other ways, like by cutting or burning”. They were told, “Remember, there are no right or wrong answers here and it isn’t always possible to help” so as not to place any guilt on participants. Items paralleled those in the eight social norms section described above. An additional item was included based on prior work, which asked about giving the person time to get better and represents a more passive response [40]. Response options ranged from 1 (*very unlikely*) to 4 (*very likely*). Missing data were no larger than 1% for each variable and recoded as the item mean. Given that the items had not been used with adolescent samples, we performed exploratory factor analyses that produced a three-factor solution. The initial principal component factor analysis using varimax rotation resulted in a three-factor solution inclusive of one item with a complex loading onto two factors (“talking to a friend about my worries”). This complex item was removed, and the factor analysis was conducted again, resulting in the final three factors. The first factor, called “use of resources” (4 items) (e.g., contact a crisis hotline for help or encourage the at-risk person to seek hotline or counseling) (α = 0.67), accounted for 31.2% of the variance and had an eigenvalue of 2.18. The second factor, called “encouragement/support” (2 items) (e.g., tell the person they matter or express worry) (α = 0.47), accounted for 17.3% of the variance and had an eigenvalue of 1.21. The third factor, called “giving the person time to get better” (1 item), accounted for 14.5% of the variance and had an eigenvalue of 1.01. Overall, the three factors accounted for a total of 63.0% of the variance. The individual complex item was examined separately in analyses and called “peer support”. One additional item was included in the table but not included in the factor analysis as it was designed to capture a range of actions and general helping intention not covered in the list provided: “I am not sure what I would do, but I would be able to help in some way”.

#### 2.1.4. Helping Behaviors

Given our interest in bystander behavior for all types of SDV exposure, we first asked whether they felt “this was a situation where it was possible for them to help this person” (yes/no). If no, we included an open-ended response asking them to say more about “why it was not possible to help this person”. If they responded yes, we asked the youth, “what, if anything, did you do to try and help this person”. Participants were reminded that there are no right or wrong answers and that it is not always possible to help. Nine specific response options were included on the survey, adapted from work by Aldrich and colleagues’ Willingness to Intervene Against Suicide Questionnaire [40] as described above. Examples included: “I talked to a friend about my worries” and “I encouraged the person to talk to their family”. Participants indicated yes or no to each of the nine (see Table 2 for a list of items). One additional open-ended item was also included at the end of this list, where participants had the opportunity to tell us how they “helped in some other way”.

#### 2.1.5. Bystander Resources

Given the common practice in gatekeeper training to encourage the person to go to someone for advice [45], we included a question that asked: “Do you have someone you can go to for advice if you are worried about a friend or family member hurting themselves on purpose?” (yes/no/not sure). We also asked whether respondents knew of a specific place, such as a hotline or crisis center, that they could share with someone that may want to hurt themselves on purpose (yes/no). For both items, not sure responses (3% and 1%, respectively) were coded as ‘0’.

#### 2.1.6. Social Support

An adapted (shortened) measure of social support [46] was included that had eight items, three referring to an adult family member and five referring to friends (e.g., “I have an adult family member who is around when I am in need”, and “My friends really try to help me”). The response options ranged from (1) *very strongly disagree* to (4) *very strongly agree*. The original scale has 12 items, including questions about a significant other, family, and friends. In the current study, we used items pertaining only to friends and family as these were two factors that were found to be most independent [46]. Missing data were no higher than 0.87% and were replaced with individual item means. Items were combined to reflect a total social support score with higher values indicating more support (α = 0.82).

#### 2.1.7. Demographic Characteristics

*Age* was captured as a continuous variable, ranging from 13–23 years. Self-reported *household income* comprised three answer choices: lower than average, about average, and higher than average. For multivariate analyses, those who indicated their family income was “lower than average” were compared to all other participants. Participants reported their *race* (coded as White vs. other, Black vs. other, and Mixed race vs. other) and *ethnicity* (coded as Hispanic versus other) separately. Gender identity options included male, female, transgender, gender queer, non-binary, pangender. All participants who indicated a gender minority option (transgender, gender queer, non-binary, pangender) were given a score of “1” on a dichotomous variable to reflect any gender minority identity. Sexual identity options included heterosexual, gay, lesbian, bisexual, questioning, queer, pansexual, asexual, other, or unsure. For the current analyses, sexual identity was coded as any sexual minority (1) versus other (0).

### 2.2. Data Analysis

To address Research Aim 1, percentages of adolescents and emerging adults who reported any exposure to SDV as well as exposure to different types of SDV (suicide attempt, suicidal ideation, NSSI) were provided. Exposure to any SDV versus none was compared across different participant demographic characteristics using chi-square cross-tabulations. Next, descriptive details of SDV incident exposure were provided for each of the three types of SDV using descriptive statistics. For Research Aim 2, four linear regressions were conducted to examine correlates of different intentions to help someone at risk for SDV (e.g., use of resources, providing encouragement/support), including participant demographic characteristics, social norms, social support, and access to resources. For Research Aim 3, we provided descriptive statistics for responses to the survey questions about the different ways participants said they helped someone they knew who was at-risk for SDV among those with opportunity; we did this for each of the three types of SDV separately and also reported percentages for any endorsement of different helping behaviors across type. Additionally, for this aim, qualitative analysis of open-ended responses to the question about what else they did to help (for the subset of participants who indicated they tried to help) and the question about why respondents felt that they could not help (for those participants who said helping was not possible) was content coded by the first author using a set of codes developed with the research team following principles of content coding [47,48,49]. Finally, to address Research Aim 4, one logistic regression was conducted examining correlates of actual helping behavior (yes/no) aggregated across all three types of SDV among those with opportunity. The amount of missing data is detailed within each construct. Missing data were replaced with the item means due to low percentages of missing items. Other techniques to replace missing data were considered but not ultimately conducted because the majority of the missing data in the study was related to participants who did not have the opportunity to intervene, which is a very different group and thus not appropriate for use in imputation.

## 3. Results

### 3.1. Exposure to SDV by Type (Research Aim 1)

More than 8 in 10 (83.1%) participants reported exposure to someone else’s SDV in their lifetimes: 53.6% knew someone who had died by suicide or made an attempt, 62.1% knew someone who was thinking about suicide but had not made an attempt, and 43.2% knew someone who engaged in NSSI. Knowing more than one person within each SDV type was common: 72.4% of participants knew more than one person who had died by suicide or made an attempt; the same was true for 70% of those with exposure to suicidal ideation, and 69% of those exposed to NSSI. Exposure to five or more people who died by suicide was reported by 13.5%, 25.3% for suicidal ideation, and 22.3% for NSSI. Participants were able to provide follow-up information on up to three unique incidents (one for each type of SDV): 31.1% of all participants reported on one type of SDV exposure, 27.6% reported on two, and 24.1% on all three. Participants could report on all three types of SDV exposure as long as different people/situations were the focus.

Chi-square tests indicated that exposure to any type of SDV was more common among emerging adults than adolescents (*p* = 0.004) (Table 1). Racial differences were noted: White participants reported more exposure compared to non-White participants (*p* < 0.001); Asian participants reported significantly less (*p* < 0.001) compared to all other races. Participants who identified with a sexual minority identity were significantly more likely to report exposure to SDV than those who identified as heterosexual (*p* < 0.001), as were gender minority participants compared with cisgender males and females (*p* < 0.001). Differences also were noted by family income: Those with a lower-than-average income reported more exposure to any SDV than those with similar or higher than average incomes (*p* < 0.001).

### 3.2. Details of the Most Recent Exposure to SDV by Type (Research Aim 1)

Within each SDV type, if participants knew more than one person who had committed or attempted SDV, they were instructed to think of the person who had most recently attempted SDV and provide detailed information about their experience (Table 3). Across all three SDV types, most commonly, respondents said that the person engaging in SDV was a close friend. Many incidents were recent: 37.4% of suicide attempts, 46.9% of suicide ideation, and 36.9% of NSSI happened less than one year prior to the survey. In many situations (58.9% of suicide attempts, 73.0% of suicidal ideation, and 51.5% of NSSI), participants said the person engaging in SDV told them about it.

### 3.3. Correlates of Intent to Help in Response to Someone’s SDV (Research Aim 2)

In previous research, the most commonly studied helping outcome is the expressed *intent to help*. In the interpersonal violence literature, intent to help in a variety of ways tends to cohere in one intent scale. However, the current data showed four distinct ways of helping in response to SDV risk. These included accessing resources (e.g., contacting a crisis hotline for help; encouraging the person to talk with their family); providing encouragement and support (e.g., telling the person you are worried about them); seeking peer support for self (i.e., talking to a friend about my worries); and giving the person time to get better. (See Table 4). Four linear regression analyses were conducted to examine how the correlates measured explained variance in each type of intent.

Social norms about helping someone at risk for SDV were consistently correlated with intent to help across all of these four avenues. Knowing a specific adult one could go to for advice was positively related to intent to help by using resources and negatively related to giving the person time to get better. Having knowledge of a specific hotline/crisis center was related to intent to utilize resources for help. Black participants were less likely than non-Black participants to say they intended to provide encouragement or to utilize peer supports; mixed-race participants were less likely to say they would utilize resources. Sexual minority participants were more likely than non-sexual minority participants to say they would ask for peer support. Both gender minority and female participants were less likely than non-gender minority and non-females to say they would reach out to get resources for themselves from a peer. Finally, social support was positively related to saying one would get peer support if one became aware of someone who wanted to hurt themselves.

### 3.4. Types of Bystander Behavior by Type of SDV (Research Aim 3)

Three in four (74.5%) of the participants who were exposed to any type of SDV said they did something to try to help. Specifically, 35.8% of those exposed to a suicide attempt, 72.2% of those exposed to suicidal ideation, and 54.6% exposed to someone engaging in NSSI tried to help (see Table 2 for the actual helping behaviors on the survey). Participants endorsed a range of helping behaviors within any one incident with an average of 5.70 helping behaviors for those exposed to a suicide attempt (SD = 1.92), 5.61 for those exposed to suicide ideation (SD = 1.67), and 5.49 for those exposed to NSSI (SD = 1.84). No significant differences were noted between adolescents and young adults for any of these helping behavior counts.

The more common helping behaviors included telling the person they were important to them and telling the person they were worried about them—an encouragement/support approach to helping. Participants also sought resources for themselves; 24.9% of those exposed to a suicide attempt, 52.2% of those exposed to suicide ideation, and 40.7% of those exposed to NSSI said they talked to a friend about their worries. It should be noted that only 22.2% of people exposed to someone engaging in suicidal behavior found out before the attempt; this may be why they have the lowest likelihood of all three exposures to report trying to intervene. This sample also had the opportunity to respond to an open-ended “other” write-in option that was content coded by the first author and a research assistant. In response to the “other” write-in option, across types of SDV helping participants talked about monitoring/checking in on the at-risk person, offering positive coping strategies, including them in one’s friend group, talking to them to let them share feelings and vent or to provide reassurance or to try to help them see the value in their life, sharing one’s own struggles, coaching them to get rid of SDV methods they had access to, prayed or connected with a spiritual leader. Participants talked about staying on the phone or being physically present “for however long they needed and used my persuasion to convince them they mattered and that their pain was only temporary and others would be really hurt by it and that she had plenty of people that truly loved her”.

### 3.5. Why It Was Not Possible to Help (Research Aim 3)

Table 5 presents a summary of the open-ended responses about why it was not possible to help. For all three SDV situations, one of the top barriers was not finding out about the SDV attempt or behavior until after it happened. Related to suicide attempts, the most common barriers were living at a distance (including only knowing the person online), not being that close to the person (assuming others would be better supports), and not seeing warning signs. One participant noted, “Because I cannot see her in person. I only Facetime her occasionally, and see her socially distanced like once a month …”. Another wrote, “This was a friend I knew online who played with and interacted with a lot over the years, but besides talking to them about how much they mattered to me, I couldn’t physically go to them and give them that conversation in person and be there for them in a way that really mattered”. Another participant wrote, “I was not an important enough person in their life to make an impact”. This was similar to the responses for suicidal ideation exposure.

For NSSI, in addition to not being close to the person, participants also identified the barrier of not knowing what to do (including the feeling that there was not anything helpful that could be done). For example, one participant wrote, “The reason was clearly private and I only wanted to address it if they brought it up. Since they didn’t, I felt it was an invasion of privacy to ask”. Another wrote, “I didn’t know what to say to them and the scars weren’t fresh and I didn’t want to get the school involved”.

For all three situations, about 14% of participants noted that the person pushed them away and refused help. One participant described someone they knew who was showing signs of NSSI: “They were not at all receptive to what I was trying to say”. Another wrote, “My attempts at help would’ve been unsolicited/unwanted, and would not have helped and instead would’ve created unhelpful tension”. Another participant, discussing trying to help someone expressing suicidal intent remarked, “Because I tried helping and they pushed me away and didn’t want help”. Discussing someone who made a suicide attempt, one participant wrote, “They didn’t want help, they said they had everything taken care of”. However, another described, “I tried my best to help them by being a good friend and offering advice, but ultimately their choices are not in my control and none of the things done for them by anyone were enough. This person still is at risk but now denies any therapy or medication because they say it makes them feel worse”. As yet another example, “She had left our house to live on her own and was refusing to speak to anyone or tell anyone what was wrong”. These responses illustrate the complexity that participants had to navigate in figuring out if they could help.

### 3.6. Correlates of Bystander Behavior for SDV (Research Aim 4)

Among those with exposure to any type of SDV, 74.5% (*n* = 638) of participants did something to try and actively help the individual engaging in SDV. Logistic regression was used to examine variables related to any self-reported overall helping. Correlates of helping were having an adult they could go to for advice (aOR = 1.60, *p* = 0.01) and having knowledge of a hotline or crisis center (aOR = 1.48, *p* = 0.02) (Table 6). Each of the four avenues for intent to help did not correlate with actual helping behavior, nor did social norms around helping in these situations.

## 4. Discussion

The current study extends our understanding of bystander intervention to help individuals engaged in SDV behaviors by including a wider age range (adolescents rather than college students or adults) and by studying actual self-reported helping behaviors in addition to intent to help. The current study shows that a high number of adolescents and emerging adults are exposed to some form of SDV in their lifetime. Older participants (ages 18–23) reported greater exposure, as did participants who listed their race as white, sexual and gender minority participants, and those living in low-income households. These findings are similar to other studies that showed greater exposure among sexual and gender minority youth and among older youth [4,37,50]. Previous research on the risk for SDV finds some differences between younger and older adolescents on factors that increase risk for suicide [51]. Previous research on interpersonal violence suggests that bystander intervention training for prevention works better for younger rather than older audiences and that prevention needs to be developmentally tailored [39]. Studies of prosocial bystanders to peer bullying show some changes with age in overall helping but also in key correlates of helping [52]. The current study did not find age was a significant correlate of intent to help or of actual bystander behavior. It was beyond the scope of the current exploratory study to examine how age might moderate the effects of different facilitators and inhibitors of helping, but this is a key question for future research.

Importantly, many participants reported knowing more than one person who engaged in SDV behavior. Consistent with previous research indicating that individuals thinking about suicide are most likely to disclose to their peers, participants most often discussed a friend in the follow-up questions and most often heard about the SDV directly from the person [7,21]. A smaller group witnessed a suicide attempt. Relatively few participants said they learned about the SDV incident online, though there were some who did; this is an understudied area that could be explored in more detail in future research with larger samples.

Participants also reported a range of helping behaviors; the most often used across the three SDV types was expressing concern to the at-risk person and telling them that they matter. A significant percentage of participants (from one quarter to one half) also took the more passive approach of giving the person time to get better. Notably, this response was less frequent for the more serious form of SDV, a suicide attempt, as compared to ideation or NSSI. Overall, ideation and NSSI elicited varied helping responses from participants. This suggests that adolescents and emerging adults may already have a skill set and positive attitudes toward intervening with these types of SDV but may benefit from more training related to responding to suicide attempts. The least endorsed behavior was contacting a crisis hotline or encouraging a person to do so, along with talking to an adult. This is notable, especially given that training programs such as Sources of Strength emphasize these helping behaviors as among the most important for peers to do [20]. The current findings reinforce the need for more widespread implementation of such programs.

The open-ended responses illustrated the range of barriers to helping that adolescents and emerging adults report. This includes access to the at-risk individual (how can one intervene if not geographically close or if only communicating online?) and suggests that prevention programs may want to highlight how helping can take place at a social distance or online (especially important in the context of the ongoing COVID-19 pandemic that was occurring when these data were collected). A number of participants indicated not seeing warning signs or not knowing how to help, another indication of the need for prevention training. Importantly, participants noted that in some cases, the person they were worried about actively rebuffed their attempts at helping. More discussion of how to help bystanders cope with these types of situations could be beneficial.

Theoretically, and consistent with the situational-cognitive model, perceptions of positive helping social norms were strongly correlated with higher expressed intent to help. This is consistent with research on bystanders and interpersonal violence prevention, where bystander models have been most often tested but also consistent with newer applications to SDV that used adult samples [14,23,28]. Having available social support for oneself as a potential bystander was also related to some forms of intent to help, suggesting that it is not only important to teach helping skills but to make sure that bystanders themselves have resources for coping and support. Demographic variation is difficult to unpack given that the current study measured only demographic characteristics and not any underlying experiences that may be connected to demographic social addresses. For example, sexual and gender minority participants were less likely to go to adults for help (perhaps indicating the varied levels of adult and family support that this community encounters), and mixed-race participants indicated less intent to seek help from external resources (perhaps due to experiences of racism or micro-aggressions when contacting organizations). Future research is needed to better understand why individuals feel comfortable with different types of helping. Surprisingly, however, this theoretical model did not hold up as well for predicting who actually helped compared to who did not. Instead, actual self-reported helping behavior was better understood in relation to specific knowledge of resources and possession of supports by the bystander. Building potential bystanders’ resources for helping may be an important component of prevention. Future research should study other strengths and resources, including school connectedness and community support as potential correlates of actual helping behaviors.

There are a number of limitations to the current study. Given that this is an understudied area measurement, development is still needed. In the interpersonal violence field, intent to help across different types of helping and situations tends to cohere as one construct. In the current study, the intent to help separated out into different forms of helping. This was a surprising finding and resulted in small subscales with few items and low reliability scores. Future research to enhance measures of intent to help that are developed from the perspective of adolescents and emerging adults is needed. The open-ended barriers items suggest a further content area for a quantitative measure of barriers to helping that can also enhance this work. In the current study, this question was only posed to participants who indicated that they did not think it was possible to help. More research is needed on a broader measure of barriers among participants who do help so that this construct can be analyzed as a correlate of helping behavior. As mentioned above, there was some significant variability by demographic variables such as gender identity, sexual minority group status, and race. It is difficult to understand these findings in the absence of a more thorough and detailed investigation of potential mechanisms for these effects. There is a growing literature using minority stress theory to understand suicide risk [53,54]. In the interpersonal violence field, experiencing victimization (which occurs disproportionately among some groups) is related to greater helping [55]. More nuanced and detailed research to unpack variation in helping behavior related to SDV is needed, particularly given that the current sample was a majority white sample. Indeed, overall, the sampling strategy produced a self-selected, social media using, and a nonrandom sample of participants. Further, the open-ended question about other forms of helping revealed a number of additional strategies, including check-ins/monitoring of the person, helping them get rid of tools they could use to implement SDV, and providing ongoing social support that should be captured in revised measures of SDV helping.

## 5. Conclusions

In spite of these limitations, the current study is one of the few studies to include and explore SDV exposure and helping behaviors among adolescents and emerging adults. The findings suggest that adaptation and revision of the popular situational-cognitive model of bystander intervention may be needed to support prevention training innovations. Programs, such as Mental Health First Aid and Sources of Strength, show promise for changing social norms to be supportive of help seeking and for promoting peer support for adolescents and emerging adults in distress [20,56]. Given a common bystander framework with interpersonal violence prevention programs, future work could investigate how such strategies might work together.

## Figures and Tables

**Table 1:** Exposure to SDV by participant characteristics.

Characteristic	All Participants (*N* = 1031) % (*n*)	No Exposure to Self-Directed Violence (*n* = 174) % (*n*)	Exposure to Self-Directed Violence (*n* = 857) % (*n*)	X^2^ *p* Value
Age				
13–17 years	67.8 (699)	77.0 (134)	65.9 (565)	0.004
18–23 years	32.2 (332)	23.0 (40)	34.1 (292)	
Race ^a^				
White	76.0 (784)	65.5 (114)	78.2 (670)	<0.001
Black	8.5 (88)	10.3 (18)	8.2 (70)	0.35
Asian	9.3 (96)	17.2 (30)	7.7 (66)	<0.001
Native American	2.7 (28)	1.7 (3)	2.9 (25)	0.38
Mixed race	10.5 (108)	7.5 (13)	11.1 (95)	0.16
Ethnicity				
Hispanic/Latino ethnicity (any race)	17.7 (183)	17.2 (30)	17.9 (153)	0.85
Sexual identity				
Heterosexual	44.6 (460)	66.7 (116)	40.1 (344)	<0.001
Sexual minority	55.4 (571)	33.3 (58)	59.9 (513)	
Gender identity				
Male	43.2 (443)	54.1 (93)	41.0 (350)	<0.001
Female	39.4 (404)	40.7 (70)	39.2 (334)	
Gender minority	17.4 (178)	5.2 (9)	19.8 (169)	
Family income				
Higher than average	20.5 (211)	21.8 (38)	20.2 (173)	<0.001
Similar to average	51.2 (528)	58.1 (101)	49.8 (427)	
Lower than average	20.9 (215)	8.6 (15)	23.3 (200)	
Not sure	7.5 (77)	11.5 (20)	6.7 (57)	
Current education status				
Middle school (6–8 grade)	15.9 (164)	20.7 (36)	14.9 (128)	0.16
High school (9–12 grade)	56.3 (581)	58.1 (101)	56.0 (480)	
High school graduate (not enrolled)	5.6 (58)	4.0 (7)	5.9 (51)	
Dropped out (stopped attending before completing high school)	2.0 (21)	1.1 (2)	2.2 (19)	
Higher education (trade or college)	20.1 (207)	16.1 (28)	20.9 (179)	

^a^ Multiple responses possible.

**Table 2:** Types of bystander behavior by type of SDV exposure among adolescents and emerging adults.

Variable	Exposure to Suicide Attempt (*n* = 553) % (*n*)	Exposure to Suicidal Ideation (*n* = 640) % (*n*)	Exposure to NSSI (*n* = 445) % (*n*)	Any Action (Regardless of Type of SDV) (*n* = 857) % (*n*)
Contacted a crisis hotline for help	5.1 (28)	7.7 (49)	4.9 (22)	10.6 (91)
Encouraged the person to contact a hotline	14.7 (81)	27.5 (176)	23.1 (103)	31.9 (273)
Encouraged the person to get counseling	25.3 (140)	52.7 (337)	36.9 (164)	55.1 (472)
Talked to an adult for help and advice about someone who is suicidal	16.5 (91)	26.6 (170)	16.6 (74)	30.9 (265)
Encouraged the person to talk to their family	19.0 (105)	36.3 (232)	25.8 (115)	41.3 (354)
Told the person I was worried about them	31.7 (175)	64.2 (411)	48.8 (217)	68.6 (588)
Told the person they are important to me	33.1 (183)	67.8 (434)	48.8 (217)	69.5 (596)
Talked to a friend about my worries	24.9 (138)	52.2 (334)	40.7 (181)	57.2 (490)
Gave the person time to get better	26.9 (149)	56.9 (364)	47.6 (212)	62.4 (535)
Helped in some other way	6.9 (38)	13.3 (85)	6.5 (29)	15.4 (132)
Any of the above	35.8 (198)	72.2 (462)	54.6 (243)	74.5 (638)

Note: Incidents are unique across SDV types.

**Table 3:** Details of the most recent exposure to self-directed violence by type.

Incident Details	Suicide Attempt (*n* = 553) % (*n*)	Suicidal Ideation (*n* = 640) % (*n*)	Non-Suicidal Self-Injury (*n* = 445) % (*n*)
Relationship with person experiencing the SDV			
Close friend	59.3 (328)	64.8 (415)	60.5 (269)
Brother or sister	9.0 (50)	9.5 (61)	5.8 (26)
Parent or other caregiver	3.4 (19)	4.7 (30)	1.3 (6)
Student in my school	12.1 (67)	8.7 (56)	20.7 (92)
Someone else	14.8 (82)	10.6 (68)	10.3 (46)
Decline to answer	1.3 (7)	1.6 (10)	1.3 (6)
Recency of event			
Less than 1 year ago	37.4 (207)	46.9 (300)	36.9 (164)
1–3 years ago	40.9 (226)	37.2 (238)	34.8 (155)
4–5 years ago	10.9 (60)	8.7 (56)	15.5 (69)
6–10 years ago	4.0 (22)	1.6 (10)	5.6 (25)
More than 10 years ago	1.5 (8)	0.5 (3)	2.0 (9)
Not sure/decline to answer	5.4 (30)	5.2 (33)	5.2 (23)
How learned about the SDV behavior			
The person told me	58.9 (326)	73.0 (467)	51.5 (229)
I saw it happen	7.8 (43)	0	0
Someone else told me	25.5 (141)	9.1 (58)	7.0 (31)
Saw marks, such as cutting	0	9.2 (59)	36.2 (161)
Online through social media	2.7 (15)	2.7 (17)	1.6 (7)
Some other way	4.3 (24)	3.8 (24)	2.7 (12)
Decline to answer/missing	0.7 (4)	2.3 (15)	1.1 (5)

Note: Incidents are unique across SDV types.

**Table 4:** Linear regression analyses examining correlates of intent to help in response to SDV (N = 1031).

	Model 1: Use of Resources			Model 2: Encouragement/Support			Model 3: Peer Support			Model 4: Time to Get Better		
	β	SE	*p* Value	β	SE	*p* Value	β	SE	*p* Value	β	SE	*p* Value
Age	0.01	0.02	0.84	−0.02	0.01	0.43	0.06	0.01	0.07	**−0.07**	**0.01**	**0.02**
Hispanic or Latino ethnicity	0.05	0.17	0.08	−0.03	0.07	0.38	0.005	0.07	0.88	−0.01	0.08	0.64
White race	−0.01	0.16	0.76	−0.03	0.06	0.40	0.01	0.07	0.71	**−0.07**	**0.08**	**0.05**
Black race	−0.04	0.24	0.22	**−0.07**	**0.09**	**0.03**	**−0.08**	**0.10**	**0.02**	0.01	0.12	0.83
Mixed race	**−0.09**	**0.22**	**0.002**	-0.02	0.09	0.60	0.04	0.09	0.27	−0.02	0.11	0.62
Sexual minority	0.01	0.13	0.78	0.01	0.05	0.65	**0.07**	**0.06**	**0.04**	0.03	0.07	0.32
Gender minority	−0.03	0.18	0.34	0.02	0.07	0.59	**−0.07**	**0.08**	**0.03**	0.03	0.09	0.32
Cisgender female	0.04	0.13	0.13	−0.003	0.05	0.91	**−0.07**	**0.06**	**0.03**	0.02	0.07	0.63
Lower than average income	−0.01	0.15	0.56	0.02	0.06	0.44	−0.002	0.07	0.95	−0.02	0.07	0.48
Social norms total	**0.42**	**0.02**	**<0.001**	**0.25**	**0.01**	**<0.001**	**0.13**	**0.01**	**<0.001**	**0.15**	**0.01**	**<0.001**
Adult you can go to for advice	**0.14**	**0.14**	**<0.001**	−0.02	0.06	0.54	0.04	0.06	0.23	**−0.07**	**0.07**	**0.04**
Knowledge of hotline/crisis center	**0.17**	**0.13**	**<0.001**	0.01	0.05	0.79	0.01	0.05	0.77	0.02	0.06	0.58
Social support	−0.01	0.02	0.82	0.004	0.01	0.89	**0.12**	**0.01**	**<0.001**	**−0.06**	**0.01**	**0.05**

Note: Bolded text highlights significance at *p* ≤ 0.05 or better. All models also adjust for self-reported honestly in answering survey questions. SE = standard error.

**Table 5:** Reasons why it was not possible to help by different types of SDV exposure.

Response type	Suicide Attempt (*n* = 319) % (*n*)	Suicidal Ideation (*n* = 142) % (*n*)	NSSI (*n* = 179) % (*n*)
Found out after the attempt	25.4 (81)	16.2 (23)	27.4 (49)
Lived at a distance/not present at time/Not in contact at the time	21.6 (69)	21.8 (31)	8.9 (16)
Not very close to the person at the time	17.6 (56)	20.4 (29)	19.0 (34)
Person kept bystander away; refused help	14.4 (46)	13.4 (19)	14.0 (25)
Too young at the time to be aware or help	6.9 (22)	5.6 (8)	5.6 (10)
Participant described causal factors that would be hard to impact with helping	6.0 (19)	7.0 (10)	4.5 (8)
Not sure what to do/Did not think anything could be done	4.7 (15)	8.5 (12)	15.1 (27)
Did not know warning signs	2.2 (7)	0.7 (1)	0

Note: Incidents are unique across SDV types.

**Table 6:** Logistic regression of correlates of helping behavior for SDV among those with the opportunity (*n* = 857).

	Odds of Any Helping		
Construct	aOR (95% CI)	SE	*p* Value
Demographic characteristics			
Age	0.97 (0.92, 1.03)	0.03	0.39
Hispanic or Latino ethnicity	1.08 (0.70, 1.69)	0.24	0.72
White race	1.02 (0.66, 1.57)	0.23	0.93
Black race	0.53 (0.30, 0.95)	0.16	0.03
Mixed race	1.09 (0.63, 1.90)	0.31	0.75
Sexual minority	1.27 (0.89, 1.81)	0.23	0.18
Gender minority	1.08 (0.68, 1.72)	0.26	0.75
Cisgender female	1.07 (0.75, 1.53)	0.19	0.70
Lower than average income	1.07 (0.73, 1.57)	0.21	0.72
Intent to help type			
Use of resources	1.03 (0.95, 1.12)	0.04	0.48
Encouragement/support	1.11 (0.90, 1.36)	0.11	0.32
Peer support	0.88 (0.72, 1.07)	0.09	0.20
Time to get better	0.95 (0.81, 1.13)	0.08	0.60
Social norms total	1.00 (0.95, 1.05)	0.03	0.97
Adult you can go to for advice	1.60 (1.11, 2.29)	0.29	0.01
Knowledge of hotline/crisis center	1.48 (1.07, 2.06)	0.25	0.02
Social support	0.99 (0.95, 1.03)	0.02	0.62

Note: aOR = adjusted odds ratio; SE = standard error. Model is also adjusted for self-reported honesty in answering survey questions.

## Data Availability

The data for this study is not publicly available.
